# AlphaZe∗∗: AlphaZero-like baselines for imperfect information games are surprisingly strong

**DOI:** 10.3389/frai.2023.1014561

**Published:** 2023-05-12

**Authors:** Jannis Blüml, Johannes Czech, Kristian Kersting

**Affiliations:** ^1^Artificial Intelligence and Machine Learning Lab, Technical University of Darmstadt, Darmstadt, Germany; ^2^Hessian Center for Artificial Intelligence (hessian.AI), Darmstadt, Germany; ^3^Centre for Cognitive Science, Technical University of Darmstadt, Darmstadt, Germany

**Keywords:** imperfect information games, deep neural networks, reinforcement learning, AlphaZero, Monte-Carlo tree search, perfect information Monte-Carlo

## Abstract

In recent years, deep neural networks for strategy games have made significant progress. AlphaZero-like frameworks which combine Monte-Carlo tree search with reinforcement learning have been successfully applied to numerous games with perfect information. However, they have not been developed for domains where uncertainty and unknowns abound, and are therefore often considered unsuitable due to imperfect observations. Here, we challenge this view and argue that they are a viable alternative for games with imperfect information—a domain currently dominated by heuristic approaches or methods explicitly designed for hidden information, such as oracle-based techniques. To this end, we introduce a novel algorithm based solely on reinforcement learning, called AlphaZe∗∗, which is an AlphaZero-based framework for games with imperfect information. We examine its learning convergence on the games Stratego and DarkHex and show that it is a surprisingly strong baseline, while using a model-based approach: it achieves similar win rates against other Stratego bots like Pipeline Policy Space Response Oracle (P2SRO), while not winning in direct comparison against P2SRO or reaching the much stronger numbers of DeepNash. Compared to heuristics and oracle-based approaches, AlphaZe∗∗ can easily deal with rule changes, e.g., when more information than usual is given, and drastically outperforms other approaches in this respect.

## 1. Introduction

Neural networks combined with Monte-Carlo Tree Search (MCTS) have become standard in many games with perfect information such as chess and Go, but have been less successfully applied to games with imperfect information (Brown et al., 2020). AlphaZero and its predecessor AlphaGo mark a breakthrough in this area by showing that it is possible to learn a game with perfect information from zero to human level (or even beyond) by combining reinforcement learning (RL) with MCTS (Silver, 2017).

However, it is not possible to apply this type of method directly to games with imperfect information, since the planning is based on a perfectly observable environment. In recent years, projects like AlphaStar (Vinyals et al., 2019), OpenAI Five (Berner et al., 2019), and Pluribus (Brown and Sandholm, 2019) demonstrated that games with imperfect information can be learned and played with great computational effort and in a short time up to the human level and beyond. One challenge of many current state-of-the-art methods is dealing with the unknown. For some, it is not clear if they converge to a Nash equilibrium or if they just aim to develop strong strategies.

To provide a flexible but strong foundation, we introduce the AlphaZero-like framework AlphaZe∗∗^1^ for imperfect information games, which allows us to easily adapt frameworks for perfect information games such as AlphaZero (Silver, 2018) and CrazyAra (Czech et al., 2020), building bridges between current successes in the field of perfect information games and the unpredictability of hidden information. To achieve this, we stay true to the model-based nature of AlphaZero and focus on the planning algorithm. We replace MCTS in AlphaZero with our own adaptation of Perfect Information Monte-Carlo (Levy, 1989) (PIMC) because, among other reasons, its memory consumption scales better with the action and state spaces than similar methods such as Counterfactual Regret Minimization (Long et al., 2010) and it remains similar to MCTS, making it easier to adapt further methods. We call our adaptation Policy Combining PIMC (PC-PIMC), merging the results of multiple searches into one policy. We also make use of a technique we call TrueSight Learning (TSL) which improves learning performance in early stages of zero-knowledge training. Brown et al. (2020) claim that methods such as AlphaZero are not sound in imperfect information environments because the value of an action may depend on the probability that it will be chosen. In chess, for example, a good move is good whether it is played or not, but in games like poker, where players can bluff, this is not the case. We tackle this problem by encoding the hidden information in the input representation within the process.

Following a model-based approach is a sensible idea for situations with imperfect information. Indeed, one may consider using a model-free approach such as DQN (Mnih et al., 2015) or DeepNash (Perolat et al., 2022). However, they arguably require more samples than model-based approaches. In turn, they are rather expensive and likely to underperform on large problems. This is where model-based approaches can show one of their biggest advantages: sample efficiency. For instance, Fujita and Ishii (2007) demonstrate this “model efficiency” for POMDPs: adding a model reduces the number of samples required to deal with imperfect information. AlphaZe∗∗, however, builds upon AlphaZero (and in turn MDPs and not POMDPs) to be more efficient, turning imperfect state information into perfect information via sampling. This makes AlphaZe∗∗ especially strong when agents have the option to gather more and more information while acting in the environment—the game becomes more and more a perfect information game. The above describes a handful of real-world situations where you don't have all the information you need to make an important decision, but you can use actions to explore and gain information that you can then use to make better decisions.

In order to evaluate AlphaZe∗∗, we looked at certain games that are considered imperfect information games with deterministic actions and that have the possibility of gaining information during the game. We used the well-known board game Stratego and its smaller variant Barrage Stratego.^2^ To this end, we introduce an open-source environment for both games, based on and compatible with the OpenSpiel collection (Lanctot et al., 2019). Furthermore, we tested AlphaZe∗∗ on Hex and its imperfect version DarkHex, for which we used slightly adapted versions of the already existing OpenSpiel versions. The empirical results show that AlphaZe∗∗ is indeed able to learn both imperfect information games from zero knowledge and beats most of the existing agents for Barrage Stratego. This clearly shows the benefit of our arguable simple approach: advantaged of and developments for AlphaZero carry over from perfect to imperfect information games. For instance, as our experimental results demonstrate, AlphaZe∗∗ easily adapts to changes to the environment such as removing actions.

To summarize, in contrast to common belief, AlphaZero can easily be lifted to imperfect information games, e.g., via sampling, resulting in a strong baseline, AlphaZe∗∗, beating four state-of-the-art baselines specialized for the imperfect information game Barrage Stratego, loosing only to P2SRO, but dealing much better with, e.g., changes to the environment. In short, AlphaZe∗∗ works surprisingly well.

We proceed as follows: First, we review related work and give a short introduction into ML for board games. Then we explain how we use our new approaches in a reinforcement learning environment. We describe the application and give a detailed analysis of the empirical results. Finally, we discuss the results and give a conclusion and an outlook on possible future work.

## 2. Related work

### 2.1. AlphaZero and Monte-Carlo tree search

In 2017, DeepMind introduced AlphaZero (Silver, 2017, 2018), a system that teaches itself games from scratch and can beat current grandmasters or world champions in games like chess, shogi, and Go. AlphaZero, showed great success in games with perfect information, demonstrating a large improvement over the dominant strategy of minimax search for so-called two-person zero-sum games (2p0s), which are defined as games with the following two conditions: |N|=2 and *u*_1_(*z*) = −*u*_2_(*z*), meaning that if one player gets a reward, their opponent has to loose the same amount. In such games, each specific information state is given a specific value that attempts to estimate the reward of that position from the player's perspective. Three years later, CrazyAra (Czech et al., 2020) was introduced, an open-source engine for training neural networks for a variety of chess variants inspired by AlphaZero. It offers several network architectures, including the mobile RISEv2 that we used in this work. Currently, it is the strongest agent in the chess variant Crazyhouse.

A key element of these successes is the combination of reinforcement learning with Monte-Carlo Tree Search (MCTS; Kocsis and Szepesvári, 2006). At each time step or move, MCTS simulates a number of move trajectories from the current game state *s*. Action selection is based on the Upper Confidence Bounds for Trees (Rosin, 2011) scheme to balance exploitation and exploration, using the current weights of *f*_θ_. The difference between MCTS and Minimax Search lies in the way these algorithms build their search trees. While Alpha-Beta Pruning, an optimized version of Minimax Search, uses exhaustive search with a limited depth, MCTS simulates sampling with a much higher depth or until a final state is reached, not using an exhaustive search, which gives a better approximation of the outcome, i.e., the reward for our agents. Looking at recent results in the Top Chess Engine Championships, which can be seen as the World Computer Chess Championships, LCZero, an open source engine similar to AlphaZero, has been able to reach or win the finals very reliably in recent years.

### 2.2. Sampling imperfect information sets for MCTS

MCTS, as mentioned previously, is a technique which has been quite successfully applied on many games with a focus on two-player zero-sum perfect information games, leading to considerable advances in this field as demonstrated on games like Go, shogi, or chess. In recent years, some adaptations were developed to use the benefits of MCTS on games and problems with imperfect information.

Perfect Information Monte-Carlo (PIMC) is an approach based on the successes of MCTS in perfect information games, trying to adapt it to imperfect information games by reducing an imperfect information game to samples of perfect information games. This process is called determinization. These samples are then analyzed by classic MCTS. Despite some criticism and theoretical disadvantages and problems, which we will also discuss in Section 4.3, it shows some success in a wide variety of domains. Such a game where PIMC is still state of the art is Bridge. In this work, we will use PIMC as the primary sampling method due to its simplicity.

Another method that tackles the problem of uncertainty is Information Set MCTS (ISMCTS; Cowling et al., 2012), which constructs a game tree with each node representing an information set instead of a specific board position. Edges correspond to actions between information sets from the point of view of the player who plays them if we treat all moves as fully observable. This makes the computation less budget-heavy and improves the decision-making process compared to other methods like determinization. Adaptations of ICMCTS, such Many-Tree ISMCTS (Cowling et al., 2015) and Semi ISMCTS (Bitan and Kraus, 2017) advance the idea of ISMCTS. In particular, Semi ISMCTS, which tries to combine the advantages of PIMC and ISMCTS, could be interesting for future work. However, due to their complexity and their distance from the classical MCTS, they contradict our idea of a simple adaptation.

### 2.3. Pipeline policy-space response oracle and DeepNash

Stratego is often cited as an example of imperfect games. Here players have no information about their opponents at the beginning, but can gather it over the course of the game and thus converge more and more to an almost-perfect information game—but more about Stratego in Section 6.1. Currently, there are two clearly identifiable methods that tackle Stratego and propose solutions. These are Pipeline Policy-Space Response Oracles (P2SRO; McAleer et al., 2020) and DeepNash (Perolat et al., 2022). P2SRO is based on the Policy-Space Oracle Response (PSRO) algorithm from Lanctot et al. (2017). It is an algorithm based on the idea of oracles, abstract entities calculating policies for a specific player given the joint policy, and it relies on the idea of empirical game-theoretic analysis. It tries to find an optimal policy through constructing a higher level meta-game by simulating outcomes for all possible matchups of all existing player policies. After that, it trains a new policy for each player via an oracle against a distribution over the existing policies, which is typically represented by an approximate Nash equilibrium obtained by a so-called Meta-Solver. A Meta-Solver is a method that gets the current payoff tensor and calculates a meta-strategy used by the oracle method to expand the policies of each player. P2SRO is an iterating process, adding more and more updated policies to each player, while using the most current policies and drawing actions from them in its decision-making process. In summary, P2SRO is a game-theoretically inspired model-free RL approach which aims to find an optimal policy by iteratively creating policies with an oracle-based approach. DeepNash is also a model-free RL approach based on game-theoretic ideas. In their work, Perolat et al. (2022) introduce a new technique called Regularized Nash Dynamics (R-NaD) which they use to control the learning behavior of DeepNash. As the name suggests, R-NaD uses regularization for learning and is based on reward transformation and dynamic systems. Other than P2SRO, DeepNash makes use of a deep neural network with four heads: one to estimate the value function and the other three to encode the policy distribution. Both P2SRO and DeepNash are guaranteed to find approximate Nash Equilibria and as such are able to exploit non-optimal strategies effectively. DeepNash as well as AlphaZero rely on self play to train their agents. It was evaluated not only against other bots but also against human Stratego players.

The primary difference between our work and P2SRO as well as DeepNash is that both approaches are model-free, which in this case means that they do not attempt to explicitly model its opponent's private game-state or the all-knowing observer's game-state. Also, neither approach relies on classical tree search, as algorithms such as MCTS are not considered scalable enough.

## 3. Background on RL and imperfect information games

### 3.1. Reinforcement learning and Markov decision processes

In reinforcement learning, we often follow the extensive form description of games and base our notation on Markov decision processes. These can be described as follows: Here N is the set of agents and H is the set of all possible trajectories in the game. W is the set of all perfect world states, while S describes the set of all imperfect information states, also called information sets. It is defined as the game state where two or more histories are indistinguishable for the player, meaning *I*(*h*) = *I*(*h*′) and $A\left(h\right)=A\left(h^{\prime }\right)$, where *I*(*h*) defines the information state and A(h) the action space after history *h*. Z is the set of all final states, i.e., all possible completed games with their progressions and as such Z⊆S. A describes the action space, where A(h) is defined as all possible actions after history *h*. A utility function *u* is defined as u:Z→ℝ, that assigns a reward to each player for a final state. I:H→N is called the identification function and specifies the player for a given history. A perfect information state or world state *w* is defined as a state in which the entire environment is observable. The transition function τ:S×A→S determines the new state *s*′ after a given move. The observations are updated after each transition.

A typical goal in reinforcement learning is to learn a value function *v*, which can be used in a planning algorithm or directly to compute the next best move. The model is described in actor-critical methods as


(1) $$\begin{array}{l} f_{\theta }\left(s\right)=\left(p,v\right) \end{array}$$

with *v* predicting the expected outcome as well as ***p*** giving a probability distribution over all legal moves of a given information state *s*. The value *v* is learned by adjusting the weights *θ* after each step.

### 3.2. Imperfect information games

Informally, a game with imperfect information is one in which players have no shared knowledge and in which parts of the observation or environment, such as payoffs or outcomes of moves, are unobservable and knowledge varies across players. These games are often described as game theoretic problems and solved accordingly. This means to goal can often be described as finding a Nash equilibrium.

A Nash equilibrium describes a joint policy $\pi^{*}=\left(\pi_{1}^{*},\ldots ,\pi_{N}^{*}\right)$ such that for any agent i∈N no better policy can be found in regard to the opponent's strategies, as such no agent has an incentive to change their strategy or action, i.e., for each player *i* the policy $\pi_{i}^{*}$ is the best response to $\pi_{-i}^{*}$, with $\pi_{-i}^{*}$ as a joint policy of all agents in N \{i}. Nash Equilibria can formally be described as:


(2) $$\begin{array}{l} V_{\pi_{i}^{*},\pi_{-i}^{*}}^{i}\left(s\right)\geq V_{\pi_{i},\pi_{-i}^{*}}^{i}\left(s\right),\text{for any }\pi_{i} \end{array}$$

In imperfect information games, game trees are often defined using so-called information sets. An information set is defined as the state where two or more histories are indistinguishable for the player, meaning τ(*h*) = τ(*h*′) and $A\left(h\right)=A\left(h^{\prime }\right)$ hold. Thus, it is not possible for the player to decide what the current state *s* is. In imperfect information games, no player has access to this perfect world state, but only to their own private as well as the public observations of it. The observations are combined into a player-specific observable state and are identical to an information set.

Some well-known approaches in this field are Counterfactual Regret Minimization (Zinkevich et al., 2007; Burch et al., 2012; Brown et al., 2019) and Fictitious Play (Heinrich et al., 2015; Heinrich and Silver, 2016).

## 4. Introducing policy combining perfect information Monte-Carlo and TrueSight learning

MCTS has been primarily developed and tested on games with perfect information such as the Chinese board game Go (Gelly et al., 2012) and is in its core a policy-optimization algorithm for finite MDPs. The combination of RL and search in the form of MCTS has not yet been successfully applied to games with imperfect information because it has theoretical weaknesses and assumptions that do not necessarily hold in partially observable environments (Brown et al., 2020) as an example they often lack the Markov property which is the key idea behind MDPs. To illustrate this, in chess you only need the current position to find the optimal move. The history of moves is not relevant to the decision. In poker, the history of past moves does play a role in finding a good move, i.e., how have the other players bet last turn. We find, however, that despite the claims of Brown et al. (2020) that MCTS should better not be used for partially observable environments, our adapted MCTS algorithm surprisingly performs on a respectable playing strength when combined with an AlphaZero-like framework.

The key idea of our work is to adapt the search algorithm. MCTS is not able to deal with uncertainties as they occur in imperfect games since it needs to know the current state of a game to work. In imperfect games we cannot always distinguish between similar states which are then grouped in information sets. However, some adaptations have been developed to take advantage of MCTS in imperfect information games, i.e., methods such as Perfect Information Monte-Carlo (PIMC; Levy, 1989), which is often called determinization, or Information Set MCTS (Cowling et al., 2012). We propose to replace MCTS within AlphaZero with a Perfect Information Monte-Carlo based method that adapts MCTS to imperfect information games by reducing an information state to samples of perfect information states. PIMC showed great success in card games like Bridge (Ginsberg, 2001), as well as other games such as Phantom Go (Borsboom et al., 2007). PIMC has some known weaknesses, particularly in convergence and decision-making, but has the advantage of being simple, fast, robust, and scalable; making it a good compromise for games with imperfect information and large state spaces. Using PIMC allows the use of further adaptations of MCTS, while its complexity is much lower than the solution of a game in terms of game theory. These advantages help us to bridge the gap between known methods for games with perfect information and their application to games with imperfect information.

### 4.1. Policy combining perfect information Monte-Carlo

Unlike the original PIMC (Levy, 1989; Ginsberg, 2001), which sums up the value estimation of all *n* samples using a scoring function for each possible move,


(3) $$\begin{array}{l} {\text{argmax}}_{a\in A}\overset{n}{\underset{i=1}{\sum }}score\left(w_{i},a\right) \end{array}$$

we first compute the complete policy for each sample and use the policy instead of the value estimation. Therefore, we call it *policy-combining*. Values are often not normalized; moreover, they do not represent a distribution. It can happen that samples in which the agent is in a better position are considered more important than situations in which the agent is in a worse position, since the values are rather high in comparison. By using the policy, on the other hand, each sample is given comparatively equal weight in the combination of samples. So in difference to Equation (3), we combine the policies of our *n* samples ***π*_*w*_1__**, …, ***π*_*w*_*n*__** and calculate the mean value for each possible move in our distribution,


(4) $$\begin{array}{l} \pi_{s}=\overset{n}{\underset{i=1}{\sum }}\frac{\pi_{w_{i}}}{n} \end{array}$$

resulting in a new policy ***π*_*s*_**. We motivate our policy combination using common ensemble techniques such as bagging. Here, we obtain higher quality results by combining the partial results of each expert.

**Figure 1:**
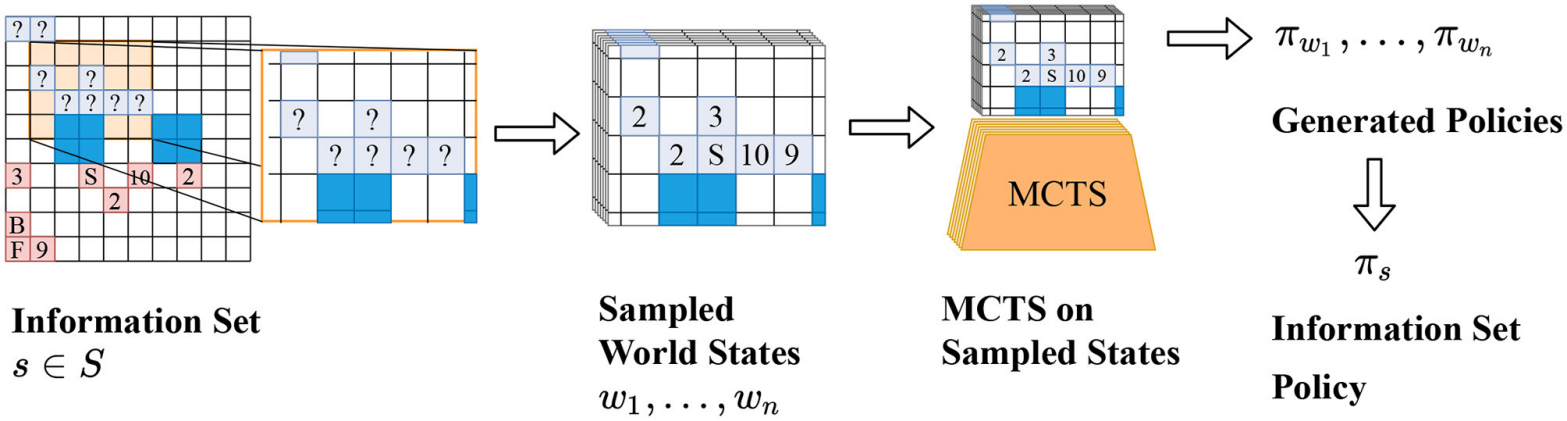
[Best viewed in color] The main principle underlying AlphaZe∗∗ is to compute a strategy for an (imperfect) information state instead of a perfect information state. From left to right: PC-PIMC first samples possible world states and then combines the resulting strategies. TrueSight Learning (not shown here) gets access to the true world state and does not sample or combine strategies at the end.

The combined policy ***π*_*s*_** can then be used to decide on the best move to play. We call this adaptation Policy Combining PIMC (PC-PIMC). It is illustrated in Figure 1 and Algorithm 1. For smaller games, it is possible to analyze an information set by applying MCTS or some other tree search algorithm to every possible state in the set. However, this does not scale well and is not feasible for larger games.

**Algorithm 1:**
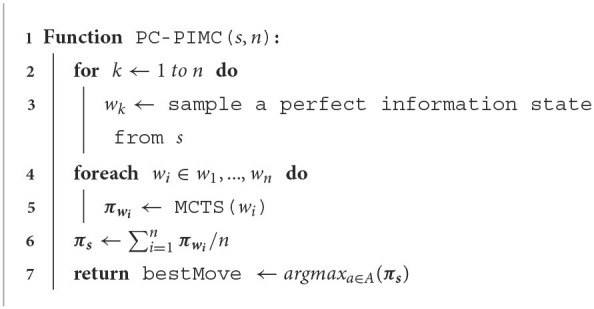
PC-PIMC with *n* samples.

The main question is how to select states from an information set in a way that is better than randomly selecting states from that set. The goal of sampling should be to prefer states that are more probable. We use the information gained while playing the game to update the probabilities over all possible states. In Stratego, when attacking a piece you get knowledge about its type, e.g., when attacking a bomb you gain the information that the enemy piece is a bomb. If you try to place a stone on an already occupied field in DarkHex, you get the information that your opponent has already placed a stone there. Together with the model of the game, we can draw conclusions about other pieces and thus optimize our sampling based on these gained information. If we get to the point where we have collected all information about the opponent and thus have perfect information, then our sampling would only contain this correct state. It is also possible to use expert knowledge, e.g., from a human expert, to optimize sampling, or to model the behavior of the opponent to get better predictions, but we omit this for now to give this paper a more concise structure.

### 4.2. TrueSight learning

Another adaptation is TrueSight Learning (TSL), a method based on PIMC and MCTS inspired by a popular human learning strategy: When people learn a new card game like Skat or poker, they often start playing with their cards face up or open to everyone, i.e., each player shows their cards face up in front of all other players. This removes the hidden information of the game and allows players to learn the basic strategies more quickly to better understand the game before playing it properly with their hands (cards) face down. This can of course be considered a form of cheating and is only used to learn a game. The basic idea is displayed in Algorithm 2.

**Algorithm 2:**
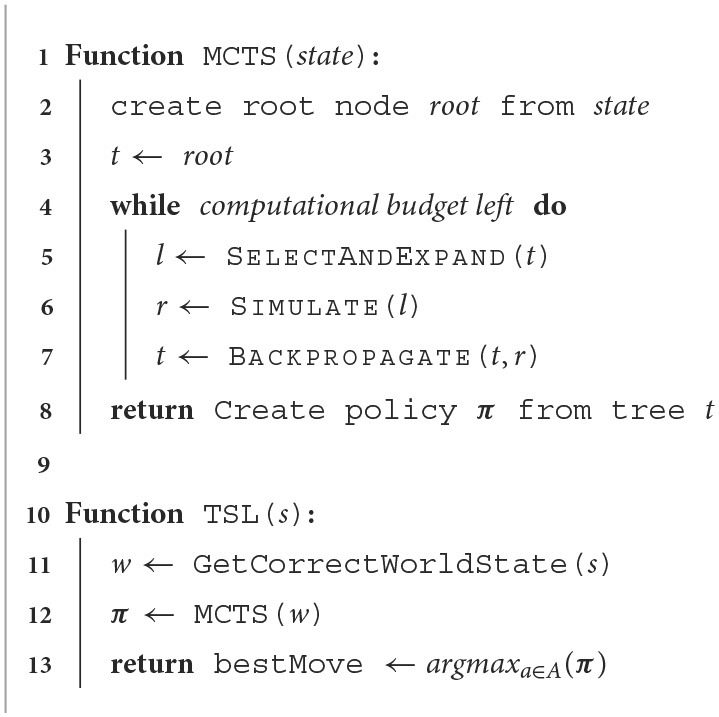
TrueSight learning.

TSL learns the game by removing the hidden information from the training process. This is similar to the PIMC approach, where the hidden information is also removed by sampling. The difference between the two methods is that TSL learns the game using only the correct, perfectly informed state, while PIMC queries probable or random states of the game that do not have to contain the correct state. The intent is to use TSL for the first epochs of training and continue the training with PIMC or PC-PIMC afterwards.

This approach aims to accelerate the initial learning process without reducing the final playing strength by giving the agent a better understanding of the game at the beginning of training. For some very difficult environments, where classical RL may not be able to learn from partial observations at all, starting with fully observable environments could help to overcome local optima and make a difference. In our experiments we used sampling-based methods like PIMC in all evaluation steps even when trained with TSL. This means TSL was only used while generating data. TSL, in principle, allows one to reuse the search results from the previous search rather than starting from scratch again. Note, however, that it is not always possible to convert partially observable environments to fully observable or to access the world state; in such cases TSL is not applicable.

### 4.3. Properties of TSL and PC-PIMC

The main concept of PIMC is to perform independent searches over different instances over multiple sampled perfect information world instances *w*_*n*_ of the information set. We presume that by increasing *n*, we get a more accurate representation of our information set. As *n* approaches ∞, our estimate of the game prediction approaches the information set *s*. However, in our evaluation, we chose a small *n* because there is a trade-off between MCTS simulations and the number of independent searches given a fixed computational budget.

However, since the introduction of PIMC, it has come under criticism. For example, the work of Frank and Basin (1998) found two different types of errors, regardless of the number of hypothetical worlds examined. First is the problem of “strategy fusion,” in which PIMC search incorrectly assumes that it can find a combined strategy that works in every world state, when in fact there are situations or information sets that must be handled differently from others. Strategy fusion imposes the constraint on a policy that it must behave the same way in all possible worlds, which is not necessarily the case in games with imperfect information.

Second, there is the issue of “non-locality” that arises from the fact that in a perfect information game, the value of a game tree node depends only on its subtree, and therefore the value of a node is determined solely by a search that starts with its children. In a game with imperfect information, the value of a node may depend on other regions of the game tree that are not included in its subtree. This is mainly due to the ability of players to steer the game toward regions of the tree that they know are advantageous to them, using private information that they possess, but their opponent does not. Long et al. (2010) examined the implications of both problems in detail and explored the conditions under which PIMC succeeds despite its theoretical shortcomings. Additionally, Long et al. (2010) gave a third potential issue but did not cover it in detail, which is the potential exploitability of PIMC search, i.e., “the performance of PIMC search could be substantially worse against a player that attempts to exploit its mistakes.”

A key difference from the original formulation of PIMC is that we use the policies instead of the value estimation. This tackles the problem of overestimating the value predictions (Wisser, 2015). However, PC-PIMC can still lead to the problem of switching between different types of strategies between moves. We call this new problem “strategy hopping” and it is mainly caused by resampling between different moves and not reusing the old search tree or samples. This remains a challenge that should be addressed in future work.

In TSL, we avoid this problem by following a strategy based on the true state of information. Nevertheless, TSL should not be applied to the entire training process, since an agent usually does not have access to the world state. Sampling the correct world state from an information set leads to a perfect information game with proven guarantees of convergence to an optimal strategy, i.e., a Nash equilibrium. Monte Carlo Tree Search based on the UCT algorithm can converge to an optimal strategy for games with perfect information, but not for games with imperfect information. Thus, while we can theoretically compute optimal strategies for each sample selected from the information set when using PIMC, we cannot guarantee an optimal strategy for the entire information set. Therefore, we cannot prove that the combination of PC-PIMC and RL converges to a Nash equilibrium, but even without such guarantees it produces strong strategies. One idea to achieve such guarantees is to replace PC-PIMC and TSL with MCCFR (Burch et al., 2012) or a special version of Information Set MCTS (Cowling et al., 2012). But even while these methods are guaranteed to converge to a Nash equilibrium, this comes with the trade-off in the form of more computation time and memory (Ponsen et al., 2011; Whitehouse, 2014).

### 5. Combining PC-PIMC with an AlphaZero-like learning architecture

Two inspirations for our work that have similar ideas to ours are called Recursive Belief-based Learning (Brown et al., 2020) and Partially Observable Monte-Carlo Planning (Silver and Veness, 2010). Recursive Belief-based Learning, which was also inspired by AlphaZero, transforms an imperfect game into an environment with perfect information by adding complexity in the form of belief states. Brown et al. (2020) use each player's private information to form so-called public belief states. The approach has beaten professional poker players in poker and has been shown to converge to approximate Nash equilibria in 2p0s games. One problem is that the search space remains large, and the algorithm has scalability problems, especially for games with little general knowledge and great strategic depth, such as Stratego. The main difference between our approach and Partially Observable Monte-Carlo Planning is that the latter builds its search tree in a brute force manner by running the search over possible “particles,” i.e., possible instances of the state. We only consider a small set of instances, which leads to better scalability for very large information sets. Moreover, our method relies on fairly large neural network approximations instead of rollout samples within the tree search, which is the case for Partially Observable Monte-Carlo Planning.

AlphaZero is a well-known model-based reinforcement learning approach, becoming a phenomenon in the world of AI. Its main advantage derives from its ability to make predictions about how a situation is likely to unfold using MCTS, i.e., it learns to predict which actions are better than others and uses this information to think ahead while staying scalable. As mentioned previously, one characteristic of AlphaZero is that it can only be used on perfect information games like chess or Go. This is mainly based in the search used within AlphaZero. The idea of this work is to introduce an easy way to adapt AlphaZero, so that we can also use it in imperfect information games, by replacing the MCTS algorithm within AlphaZero with the PC-PIMC approach introduced earlier. The remaining elements of AlphaZero, such as the policy and value network as described in Equation 1 stay the same and are used within the tree search as they would be used in MCTS. This, of course, does not solve all problems AlphaZero has with imperfect information games. It even generates new ones such as the strategy fusion problem described in Section 4.3, but it is also a first step in making AlphaZero interesting for imperfect information games. In this section, we introduce AlphaZe∗∗, a new algorithm that combines AlphaZero with PC-PIMC. To our knowledge, this is the first model-based reinforcement learning approach for games with imperfect information based on AlphaZero. AlphaZe∗∗ is intended as a baseline to show that AlphaZero-like approaches can also be interesting for imperfect games. The goal is not to create an approach specialized for imperfect information games; instead we try to stay as close as possible to the actual AlphaZero. Accordingly, the training process of AlphaZe∗∗ remains as close as possible to that of AlphaZero and is described in Algorithm 3. Learning by self-play is essentially a policy iteration algorithm. Each iteration uses a fixed amount of samples, while each sample consists of a state *s*_*t*_, the policy played in this state ***π*_*t*_** and the reward *r*_*t*_. To generate these triplets, we take our current model and play games against our self, using PC-PIMC, PIMC, or TSL. After each game we use the terminal reward *z* or in other words the reward of *s*_*t*_ while $s_{t}\in Z$ for each action played in the game. After generating a sufficient amount of data, we update our current model, using the loss function (Equation 5) and stochastic gradient descent. The only difference is replacing MCTS with (PC-)PIMC. This is the simplest possible change to AlphaZero to extend it to imperfect information games. This simplicity is one main advantage of AlphaZe∗∗, and allows us to reuse the core concepts of one of the most successful and best known algorithms for perfect information games.

**Algorithm 3:**
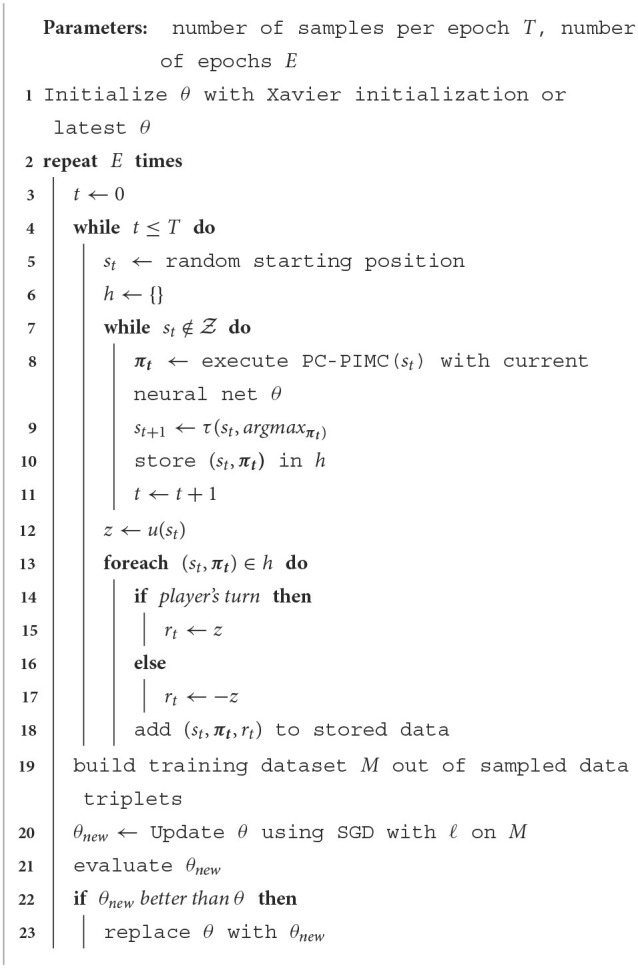
AlphaZe∗∗'s trainings process.

AlphaZe∗∗^3^ uses the CrazyAra engine of Czech et al. (2020) as a basic framework and trains a model *f*_θ_ based on the following loss function


(5) $$\begin{array}{l} \ell =\alpha {\left[z-v\right]}^{2}-\pi^{⊤}\log p+c\cdot ||\theta |{|}_{2}^{2}, \end{array}$$

which calculates a combined loss for both network outputs, where [*z*−*v*]^2^ is the mean square error between the correct value, i.e., the outcome of the game, *z* and the predicted value *v*. ***π***^⊤^log ***p*** is the cross entropy of the correct policy vector ***π*** and our predicted one ***p***. At the end we add a regularization constant *c* for our *L*_2_ weight regularization. The loss function is identical to that of AlphaZero. In addition, we also use an α value of 0.5 and a regularization term *c* of 10^−4^ as suggested in the work of Silver et al. (2016). The weights are fitted using stochastic gradient descent,


(6) $$\begin{array}{l} \theta_{t+1}=\text{SGD}\left(\theta_{t},\alpha_{t},\ell_{t}\right) \end{array}$$

and training is based on data generated by self-play. While playing, the agent takes the current imperfect information state as an input and if it is not a terminal state, it uses PC-PIMC, as described in Figure 1 to sample possible states from the current input and runs MCTS combined with the neural network, like described in the work of Silver et al. (2016). The resulting policies are combined to form a new policy, that is used to select an action to play in the current state.

There are various factors which allow scaling our approach to different circumstances, such as time limits or different board sizes. These factors include linear ones such as the amount of residual blocks of the neural network that limit the expressiveness of our model, the number of MCTS simulations during a single search that define the quality of the search, the number of sampled world states which define our approximation of the true information set. Additionally, there are quadratic factors such as the board size that influence the inference speed of our neural network.

## 6. Learning Stratego and DarkHex

In this work, we decided to use the board games (Barrage) Stratego and DarkHex as environments. Both games are well-known 2p0s games with imperfect information. We chose Stratego because of its undeniable complexity, far exceeding that of Go or chess, and as a challenge to ourselves. Two recent works have also used Stratego as an environment; the Pipeline Policy Space Response Oracle (P2SRO) approach (McAleer et al., 2020) and DeepNash (Perolat et al., 2022), which was developed in parallel with our work. Both works highlight the challenge and impact of learning Stratego, and also show that most Stratego agents currently in use are based on heuristics. As a second environment, we chose DarkHex, an imperfect version of the board game Hex. While AlphaZero has been successfully applied to Hex, there are no AlphaZero approaches for DarkHex. DarkHex also has a different kind of imperfection than Stratego: while in the latter the opponent's pieces are visible even if it is not clear what type each piece is, in DarkHex you do not see the opponent's actions at all. One advantage of DarkHex over Stratego is that it is easier to control the complexity of the game by scaling the board; also the overall complexity is less than that of Stratego. We did not choose poker, even though it is an excellent example of imperfection and playing around information, because we tried to stay close to games where AlphaZero has shown its strength, i.e., 2p0s, turn-based board games.

### 6.1. Stratego

In Stratego both players can observe information while playing, i.e., common knowledge and player specific information. We assume perfect recall, i.e., when players recover information, they never forget it. Each player has 40 pieces with different ranks or types. At the beginning of the game, each player places their own pieces, while they remain hidden from the opponent. However, in this work, we do not consider the setup phase of pieces, and rather sample random starting positions generated from human Stratego games.^4^ This data however was not used in the training process, only to set up the board.

The player who has their flag piece captured loses the game. The game is played on a 10 × 10 board with two 2 × 2 lakes as obstacles which are not passable. Stratego has a game-tree complexity of 10^535^, an average game length of 381 moves and a branching factor of ~21.739 (Arts, 2010). The board for the Barrage Stratego subvariant is identical, only the amount of pieces is reduced to eight per player.

As our game environment, we use an own implementation of Stratego based on the OpenSpiel framework (Lanctot et al., 2019). This has the advantage of being easily reusable for researchers. Furthermore, the combination with CrazyAra leads to a fast runtime due to its optimized C++ implementation of AlphaZero-like systems.

#### 6.1.1. Stratego notation scheme

**Figure 2:**
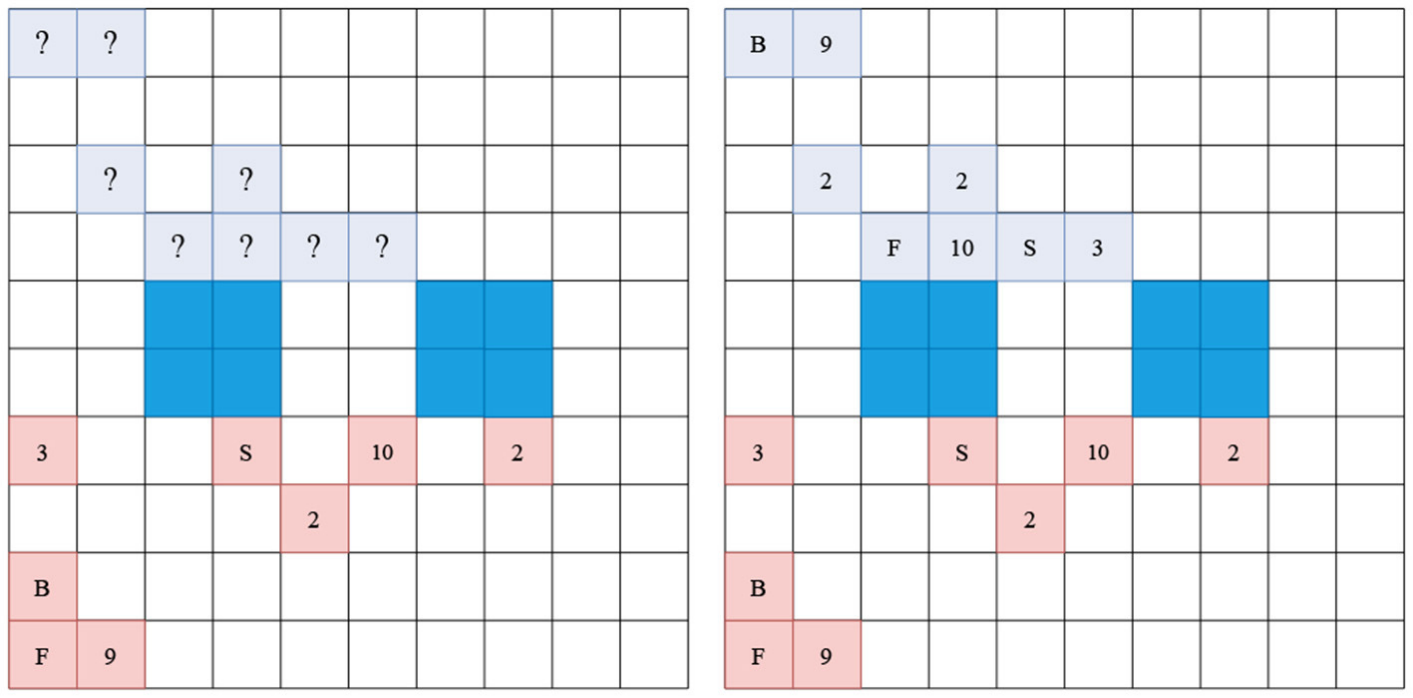
Valid Barrage Stratego starting position. The notation scheme can be found in Table 1. Pieces with a higher rank capture lower pieces on contact. Blue fields indicate lakes and are non-passable for both players. Left showing the perspective of the red player, while right shows the observer perspective of the board. The difference is in that red cannot observe the ranks of the blue pieces.

There is no official notation for Stratego because games are mostly not recorded in any form. A possible notation scheme that can be found online is called Stratego Documentation-System Version 2 (StraDoS2) and is used by a website called Gravon to notate Stratego online games, shortly described in the work by de Boer et al. (2008). The StraDos2 scheme is used to notate a game's starting position giving every piece a letter, as described in Table 1 and add the move history onto it. In this form, it is not possible to describe a position within a game without specifying the move history. For this reason, a new notation style called FEN-for-Stratego (FENfS) is introduced, which adds additional information like the current player, the move number and which pieces were involved in a fight and are thus common knowledge. The new scheme combines Strados2 and the well-known Forsyth-Edwards Notation (FEN)^5^ to display board states and piece configurations in chess and includes the following three essential parts: Firstly the piece configuration, secondly the current player and thirdly, the current move number. In the piece configuration string, each piece is displayed with the StraDos2 character. If the character of a piece is a lowercase letter, it is visible to both players; if it is uppercase, the rank is hidden from the opponent. The observer FENfS description of a position is not available to the players, containing private knowledge of the players but can be adapted by adding “?” and “!” to the view of a player. This makes it possible to display that a piece has moved, but the rank is still hidden (“!”), while pieces not touched are displayed by '?'. The information is lost if you only have the observer FENfS String. Our new scheme allows us to specify an in-game position without needing to specify the move history. The position in Figure 2 can be described as:

“BKaaaaaaaaaaaaaaaaaaaDaDaaaaaaaaMLCEaaaaaa__aa__aa

aa__aa__aaQaaOaXaPaaaaaaPaaaaaNaaaaaaaaaYWaaaaaaaa r 0"

The same position from the perspective of the red player:

“??aaaaaaaaaaaaaaaaaaa?a?aaaaaaaa????aaaaaa__aa__aa

aa__aa__aaQaaOaXaPaaaaaaPaaaaaNaaaaaaaaaYWaaaaaaaa r 0"

**Table 1:** Notation scheme for each piece within StraDos2 and FENfS.

**Piece**	**Blue**	**Red**	**For visualization**
Bomb	B	N	B
Spy	C	O	S
Scout	D	P	2
Miner	E	Q	3
Sergeant	F	R	4
Lieutenant	G	S	5
Captain	H	T	6
Major	I	U	7
Colonel	J	V	8
General	K	W	9
Marshal	L	X	10
Flag	M	Y	F
Empty field	A		
Lake	_		Dark blue

A position is displayed as a string of 100 characters, representing each field, starting with the square a1, then a2 and so on. The notation scheme also specifies a symbol used in visualizations like Figure 2.

### 6.2. Hex and DarkHex

**Figure 3:**
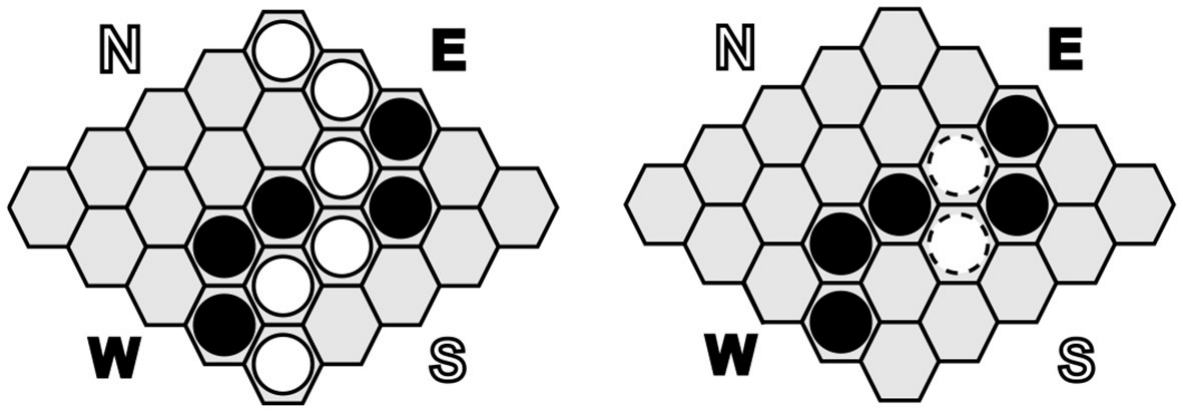
A 5×5 Hex board, showing a terminal position with white as winner in Hex on the **(left)**. On the **(right)** side a similar position in DarkHex from the perspective of the black player where two white stones have been found by black. In our work, we use a board size of 11×11.

Hex and DarkHex are played on a 11×11 board with hexagonal squares; exemplary positions are shown in Figure 3. The object of the game is to lay a row of tiles connecting two opposite sides of the board. Both players have different sides to connect, i.e., player one's goal is to connect north and south, while their opponent tries to connect west and east. In each round, a player can discard one stone of their own color. As mentioned earlier, the difference between Hex and DarkHex lies in what the players can observe. While Hex itself is a perfect information game, in DarkHex a player cannot observe the opponent's actions and only receives the information that the opponent has performed an action. If a player wants to place a piece on a square that has already been captured, they receive the information that the square is not available. There are two ways to proceed in this situation. One ruleset allows the player to choose another square to place their stone in this scenario. The second option, which we used in our experiments, has the player lose their turn if they try to place a stone on a square that is already blocked. This option is called abrupt DarkHex.

## 7. Input and output representation of AlphaZe∗∗

Similarly to AlphaZero or CrazyAra, AlphaZe∗∗ represents the game state in the form of a stack of so-called levels or planes. Each layer can be compared to a channel describing one of the input features in the current state. Each level is encoded as a map the size of the field, i.e., it contains one value of information for each field. We distinguish between three types of planes: Probability planes which have a value between 0 and 1 for each field of our board, binary maps with a value of 0 or 1 per field, and scalar planes which share a value between 0 and 1 over all fields. As an example, we can look at the first 24 planes of the input representation for Stratego (Table 2). In Stratego there are 12 different types of pieces and two players. Each of these 24 planes represents a combination of figure type and player, e.g., all scout figures of the first player. In this example, we would now have a value of 1 in just these places and 0 in all other places. A good visualization using chess as an example can be found in the work of Czech et al. (2020).

**Table 2:** Plane representation of an information set *s* in Stratego and DarkHex.

**Feature**	**Plane**	**Representation**
**Stratego**		
Pieces	1–24	Probability plane
Empty fields	25	Binary plane
Blockades (Lakes)	26	Binary plane
Unknown pieces P1	27	Binary plane
Unknown pieces P2	28	Binary plane
Side to move	29	Scalar plane
Repetition count	30	Scalar plane
**DarkHex**		
White pieces	1–3	Binary plane
Empty fields	4	Binary plane
Black Pieces	5–7	Binary plane
Side to move	8	Scalar plane

The features are encoded as planes. Each plane has one value per square of the game board describing the current game state. Binary Planes, also called tensors, have one bit per value. Probability planes describe a value between 0 and 1 per square and Scalar planes have a single value between 0 and 1 set over all values of a plane.

The difficulty with Stratego is that you only know the types of your own pieces and a few of your opponent's pieces. For this reason, in this work we decided not to represent the opponent's pieces by 0's and 1's, but instead to use floating point numbers to represent the probabilities of the piece types. Currently, the probability of each piece type is calculated as a discrete uniform distribution. These probability levels are used to find samples from a set of information in PC-PIMC sampling. The probabilities are adjusted for knowledge gained after each round, e.g., if we caught the last scout, we now know there are none left, so the probability that another piece has the scout type is 0.

The probability planes are a major difference to perfect information games, where piece types and positions can be observed perfectly and the use of probabilities is not required.

Furthermore, additional features like the repetition count or the current player are defined as an input feature. The complete stack of planes can be found in Table 2. We also add two planes, describing which pieces are currently still hidden and not common knowledge. These planes are retained even after the sampling to show which pieces are sampled and which are publicly observable. This can help the model learn to play around information, e.g., hide specific pieces from the opponent, which would not be possible if we used only PC-PIMC and the piece planes to describe the current state of private and public information. The values of each plane are scaled to the range of [0, 1] and the board is always flipped to the view of the current player.

In Hex and DarkHex there is only one piece type and the probabilities of fields being occupied are uniformly distributed. Unlike in Stratego, we do not use probability planes in the input representation of Hex/DarkHex because the only information we could use to calculate a probability plane are heuristics or other forms of human knowledge. Since we do not want to include expert knowledge, a probability plane is only a uniform distribution over all hidden fields on which the generation of samples is then based. The input planes for Hex can be seen in Table 2. We copied and adapted the representation used by OpenSpiel (Lanctot et al., 2019) for Hex and DarkHex slightly, adding the current player. As mentioned earlier, the goal of Hex is to connect two borders of the board, so each player's pieces are represented with three planes, one for each of those borders, including all pieces connected to the respective border, and a third plane for all played stones not connected to either side.

The output of AlphaZe∗∗ is described as the expected utility of a game position, represented by a numeric value in the range of [−1, 1], often called value, and a distribution over all possible actions, called policy. The size of the policy vectors differs between Stratego and Hex. In the latter, we have only the possible moves for the board height × the board width, i.e., the number of squares where a stone can be placed. In Stratego we have to count every possible move for every type of piece on every possible square. In our experiments, we computed an upper bound on the number of possible/legal actions with 3,600 actions [4 (directions) × 9 (maximum length of a move) × 100 (possible squares)]. These 3,600 actions serve as a fixed parameter defining the length or our policy. When evaluating the policy to determine a move, we only consider legal moves for the current state, i.e., we ignore illegal moves for the situation.

## 8. Deep neural network architecture of AlphaZe∗∗

As for the neural network architecture, we use RISEv2-mobile as introduced in Czech et al. (2020). The model architecture is a shared convolutional value policy network. The convolutional stem consists of 13 inverted residual blocks as introduced in MobileNet v2 (Sandler et al., 2018). Each block consists of group depthwise convolutions, batch-normalization and Squeeze Excitation Layers (SE; Hu et al., 2018). The SE Layers use a ratio *r* of two and are applied to the last five residual blocks. The number of channels for the 3×3 convolutional layer of the first block start with 128 channels and is increased by 64 for each residual block reaching 896 channels in the last block as recommended in the Pyramid-Architecture (Han et al., 2017). Finally, the convolutional stem is followed by a value and policy head which is adapted to the usage of our Stratego policy representation. For detailed information, please refer to Table 3 and Czech et al. (2020).

**Table 3:** *RISEv2 mobile* (13×256) adapted for Stratego and introduced in Czech et al. (2020).

**Layer name**		**Output size**		**RISEv2 mobile 40-Layer**	
conv0 batchnorm0 relu0		256 × 10 × 10		conv 3 × 3, 256	
res_conv0_x res_batchnorm0_x res_relu0_x res_conv1_x res_batchnorm1_x res_relu1_x res_conv2_x res_batchnorm2_x shortcut+output		256 × 10 × 10		$\left[\begin{array}{c} \text{(SE-Block, }r\text{ = 2)}\\ \text{conv 1 }\times \text{ 1, 128 + 64 }x\\ \text{dconv 3 }\times \text{3, 128 + 64 }x\\ \text{conv 1 }\times \text{ 1, 256} \end{array}\right]\times \;13$	
Value head	Policy head	1	3, 600	Value head	Policy head

## 9. Results

Before moving on to our empirical evaluation and results, let us discuss the experimental setup and training process of AlphaZe∗∗.

### 9.1. Experimental setup

#### 9.1.1. Hyperparameter settings and configuration

Our hyperparameter setup is very similar to the settings proposed in Czech et al. (2020). One difference between the proposed settings and our setup is that we do not add Dirichlet noise or temperature, which is a scaling factor applied to the posterior policy to add some randomness over the initial moves in order to improve the veracity of the training data. In Stratego, we have sufficient randomness from the randomly chosen starting positions for both players so that we do not need to add any additional noise over the policy. In PC-PIMC and TSL, after the sampling step, we use a common version of MCTS that follows the PUCT formula as proposed by Silver (2017). For PIMC we use the value head of our network as scoring function, basically getting the value of the state after playing an action and follow Equation (3).

We also introduce three new hyperparameters for our approach; namely the number of samples used for the PC-PIMC approach, the number of epochs using TSL before switching to PC-PIMC, and whether the budget within MCTS is shared among all samples or whether each sample has its own budget. For Barrage Stratego, we tested sample sizes between 1 and 10 and stuck with a sample size of 3. For DarkHex, we run experiments with various sample sizes, while the number of TSL epochs depends on the experiment. Overall we ran experiments from 5 to 50 iterations. A split budget can improve fairness and reduce computation time, but also weakens the agent and reduces the number of tree nodes (nodes) explored in each sample. A node represents a position that has been evaluated in the search process and the amounts of nodes represent the size of the MCTS tree. Unless otherwise specified, we chose not to split the budget and give each agent 800 nodes per turn. We also tested setting a fixed time limit when playing in tournament mode.

In further evaluating the playing strength of our DarkHex agents, we took advantage of the fact that DarkHex is scalable over the size of the game field. For the experiments, we used the following sizes: 3×3, 5×5, 8×8, and 11×11. The size 3×3 is used to test if our agents converge, 5×5 and 8×8 for studies around playing strength and to evaluate the effects of the number of samples per turn, 11×11 to study scalability and the evolution during the training process. Since DarkHex is smaller in size, we could use higher amounts of samples per turn.

#### 9.1.2. Training data and process

For training our approaches, we adopted a zero-knowledge approach, which means that we did not use any data to train our agents other than self-generated data. A minor exception are the starting positions for Stratego, which we took from a public dataset, as mentioned in Section 6.1. However, we decided against using this data for the training.

To generate our data, we used a technique called self-play. The training process can be seen in Algorithm 3 and is explained in Section 5. Depending on the game and experiment, we used between 409,600 and 819,200 samples per iteration.^6^ The experiments were run on 3 Nvidia Tesla V100 GPUs.

#### 9.1.3. Elo as metric

Davis et al. (2014) showed that in many large imperfect information games the computation of a Nash equilibrium is not tractable and measuring the deviation from it is not a good measurement for the quality of an agent in all cases, e.g., they showed that a more exploitable agent is able to beat a less exploitable agent in some situations. Furthermore, they argue that calculating the exploitability can become a problem in large games. We instead measured the playing strength and training development of our agents by competing with existing agents and use Elo as metric.

Elo is defined by the following equations approximating player strength based on played games:

(7) $$\begin{array}{l} R_{A}^{\prime }=R_{A}+K\left(S_{A}-E_{A}\right) \end{array}$$

(8) $$\begin{array}{l} E_{A}=\frac{1}{1+10^{\left(R_{B}-R_{A}\right)/400}} \end{array}$$

*R*_*A*_ is the current rating of agent *A*, $R_{A}^{\prime }$ the new rating, *S*_*A*_ the true score of *A* in the tournament or in competitions, *E*_*A*_ the expected score of *A* in the tournament. *K* is a regularization constant; set to 1 in our work. We initialized each agent with the same rating and measured the Elo difference between agents as an indicator of playing strength. Since all models use the same initialized starting model, we often use this to compare against.

### 9.2. Experimental evaluation

**Figure 4:**
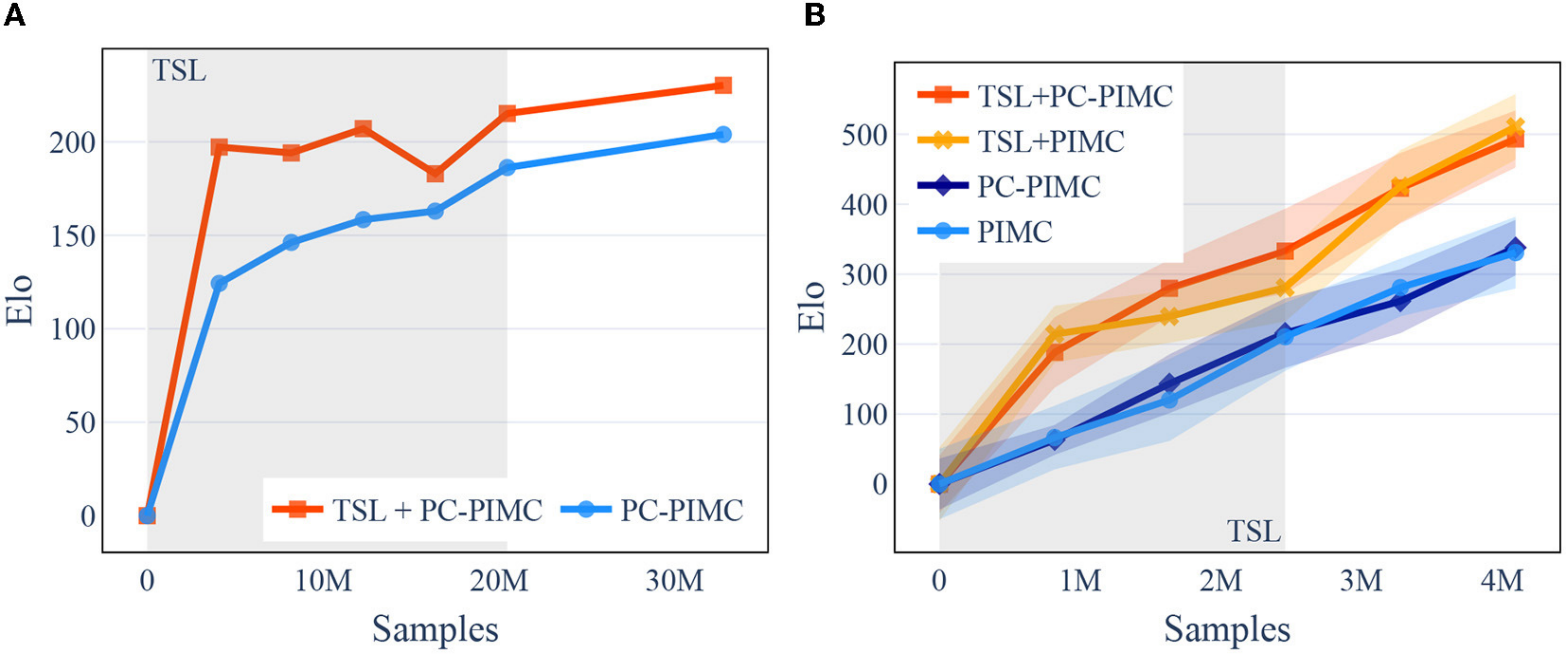
[Best viewed in color] TrueSight Learning is able to beat earlier versions of itself more often than PIMC in the early stages of learning in both Stratego and DarkHex. For the TSL models, the first n epochs (indicated in gray) were played with TSL support before switching to the second training method. Barrage Stratego uses three samples per turn, DarkHex 12 samples per turn. **(A)** Barrage Stratego. **(B)** DarkHex 11×11.

**Figure 5:**
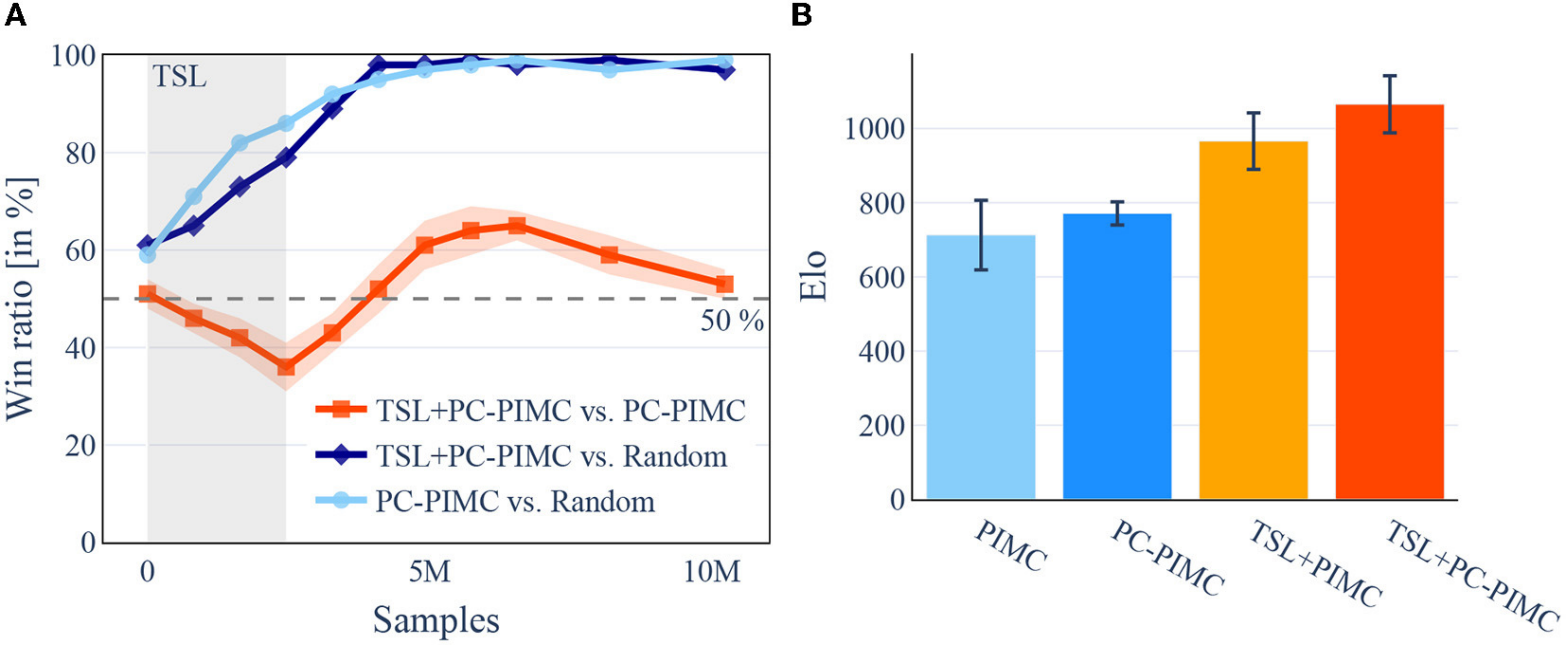
[Best viewed in color] After switching methods and a short acclimation phase, introductory training using TSL leads to better results. In turn, the use of TSL during the TSL phase shows worse results. A comparison of TSL, PC-PIMC, and PIMC in DarkHex on an 11×11 board, also illustrates the benefit of using TSL. On the left side, TSL+PC-PIMC, PIMC, and Random playing small tournaments against each other during training over 25 epochs. On the right side, we played a round-robin tournament between the agents from Figure 4B after finishing 10 epochs. All agents are using 12 samples per turn. For each comparison 100 games where played between the competitors. **(A)** Comparing win ratios during training, in DarkHex 5×5. **(B)** Round-Robin tournament, in DarkHex 11×11.

To evaluate if our agents are able to learn the games of Barrage Stratego and DarkHex, we had round-robin tournaments played between the model and its predecessors and measured the relative Elo increase, meaning models with a higher Elo were better at beating their predecessors. We trained our Barrage Stratego model 40 epochs, i.e., over 32 million training samples, and 25+15 epochs for the TSL+PC-PIMC model, meaning training the model for 25 epochs with TSL support and for 15 without. We used three samples per turn. The training took about 16 days, each epoch about 10 h. DarkHex was trained on the same architecture with 3 h per epoch and only 409,600 samples, using a board size of 11×11. We trained for 10 epochs and 6+4 for the TSL models, with a little more than four million samples in total. This took around 26 h of computing. In DarkHex we used 12 samples per turn. Due to the training costs, the experiments regarding Stratego were performed only once. The experiments to determine the graphs in DarkHex were performed three times each with different seeds to investigate the aspect of robustness and reproducibility. As we can see in Figure 4, the agents are consistently able to beat older versions of themselves, as well as beating an agent playing with a random move strategy with increasing probability, shown in Figure 5A—both indicating that they are able to learn the concepts of both games.

Our second experiment assesses the playing strength of our models. In case of Barrage Stratego against existing Barrage Stratego bots. For this purpose we played head-to-head matches of 100 starting positions, each played twice with alternating colors. We evaluate the development of our agents in relation to the number of nodes within the tree planning of AlphaZe∗∗ as well as in relation to time. We find that AlphaZe∗∗ beats current Barrage Stratego bots with an average win ratio of 36% when the amount of nodes per sampled MCTS tree (nodes) is limited to 800 but achieves an average win ratio of 59.6% (over 70% if we exclude P2SRO) when playing with 25,000 nodes per turn. As opponents, we used Asmodeus, Celsius, Celsius1.1, and Vixen which were submitted in an Australian university programming competition in 2012 and are open source^7^. They can play Barrage as well as regular Stratego. Note that these agents were not used in training as we only used self-play there. Thus, all strategies played by these agents are new to AlphaZe∗∗. All of these agents are based on handwritten rules and/or heuristics, often playing high-risk strategies. Furthermore we compared our agents against one of the current state-of-the-art methods, P2SRO. AlphaZe∗∗ is not able to reach the level of P2SRO, as can be seen in the head-to-head comparison, where it looses over 80% of its games. The complete results can be seen in Table 4.

**Table 4:** AlphaZe∗∗ outperforms Barrage Stratego agents after 50 training iterations.

**Agent**	**Amount of nodes per tree**		**P2SRO**
	**800 nodes (%)**	**25,000 nodes (%)**	
Asmodeus	**58**	**74**	**81**
Celsius	38	•**72**	**70**
Celsius 1.1	34	**68**	**69**
Vixen	46	•**68**	**65**
P2SRO	4	16	–

Shown are the win ratios (as percentage) of AlphaZe∗∗, respectively. P2SRO against common Stratego agents. For Stratego, 25 starting positions are played, against each agent, each twice with alternating colors. Bold values denote ratios where AlphaZe∗∗ resp. P2SRO are better. Ratios, where AlphaZe∗∗ outperform P2SRO are indicated by •.

Several subvariants exist for Stratego, such as the OpenStratego variant without hidden information and the Informant Spy Variant, where both players gain additional information about the opponent over time. This leads us to investigate the performance on rule changes of Stratego. For our evaluation, we examine, among other things, how the agents react when the target pieces of the game, i.e., the flags, are revealed from the beginning. This scenario can be transferred to many real-world problems where the goal is clear but the path and other circumstances are not. The first variant we introduce is called open pieces. In these variants a specific piece type, mostly high-value piece types, are openly observable for both players from the beginning of the game. These open pieces often change player strategies completely, e.g., if you know where the enemy flag is, you do not have to search for it. Of course, since we want to show the robustness to rule changes, we do not retrain or optimize our models on these variants and use the same models as in the experiments of Table 4, specifically the 25,000 node versions of it. The second variant we want to introduce is called Blocked Fields, in which we have made 10 fields inaccessible for both players, similar to the lakes in the middle of the board. Note that while these variants are inspired by variants like OpenStratego, they are not official Stratego versions and were developed by us. The results of these experiments can be seen in Table 5.

**Table 5:** AlphaZe∗∗ excels in situations when more information is provided than usual or when some fields are blocked.

**Agent**	**Open pieces**		**Blocked fields (%)**
	**Flag (%)**	**Marshall (%)**	
Asmodeus	62 ⇓	76 ⇑	78 ⇑
Celsius	84 ⇑	78 ⇑	78 ⇑
Celsius 1.1	86 ⇑	82 ⇑	72 ⇑
Vixen	60 ⇓	72 ⇑	66 ⇓

It should be noted that neither of these agents have been explicitly optimized or developed for these (new) subvariants of Stratego. ⇑ / ⇓ denotes ratios where AlphaZe∗∗ is better/worse compared to the results in normal Barrage Stratego (Table 4).

Regarding the DarkHex agents, we started by examining their playing strength. For this, we investigated how the playing strength evolves over the course of training and how the use of TSL and PC-PIMC affects it. As baseline we used an agent with random move selection. The results can be seen in Figure 5A and show that TSL+PC-PIMC is loosing to PC-PIMC in a direct comparison in the beginning, but after 10 epochs (round about 4.1 M samples), it gains the upper hand. We also see that after training is complete, TSL agents have a slight advantage.

If we run the same experiments on a 3×3 field, we can see that all sampling methods from Figure 4B manage to produce agents that converge and whose strength is comparable to each other. A difference in playing strength between methods with and without TSL cannot be observed. Only the time until convergence of the agents differs slightly in favor of the TSL agents.

For the experiments in Figure 4, we also tried to determine the playing strength by having the final models compete against each other. In Barrage Stratego, the TSL+PC PIMC wins against the PIMC model in 55 out of 100 games. Both models achieve similar win probability against an agent with random action selection, i.e., 96% (TSL+PC-PIMC) and 94% (PC-PIMC). For DarkHex, we played a round-robin tournament with the agents from Figure 4B and then determined the Elo, where 0 represents the Elo of the random agent. The results are shown in Figure 5B. In both experiments, the TSL agents perform slightly better than the agents without TSL.

**Figure 6:**
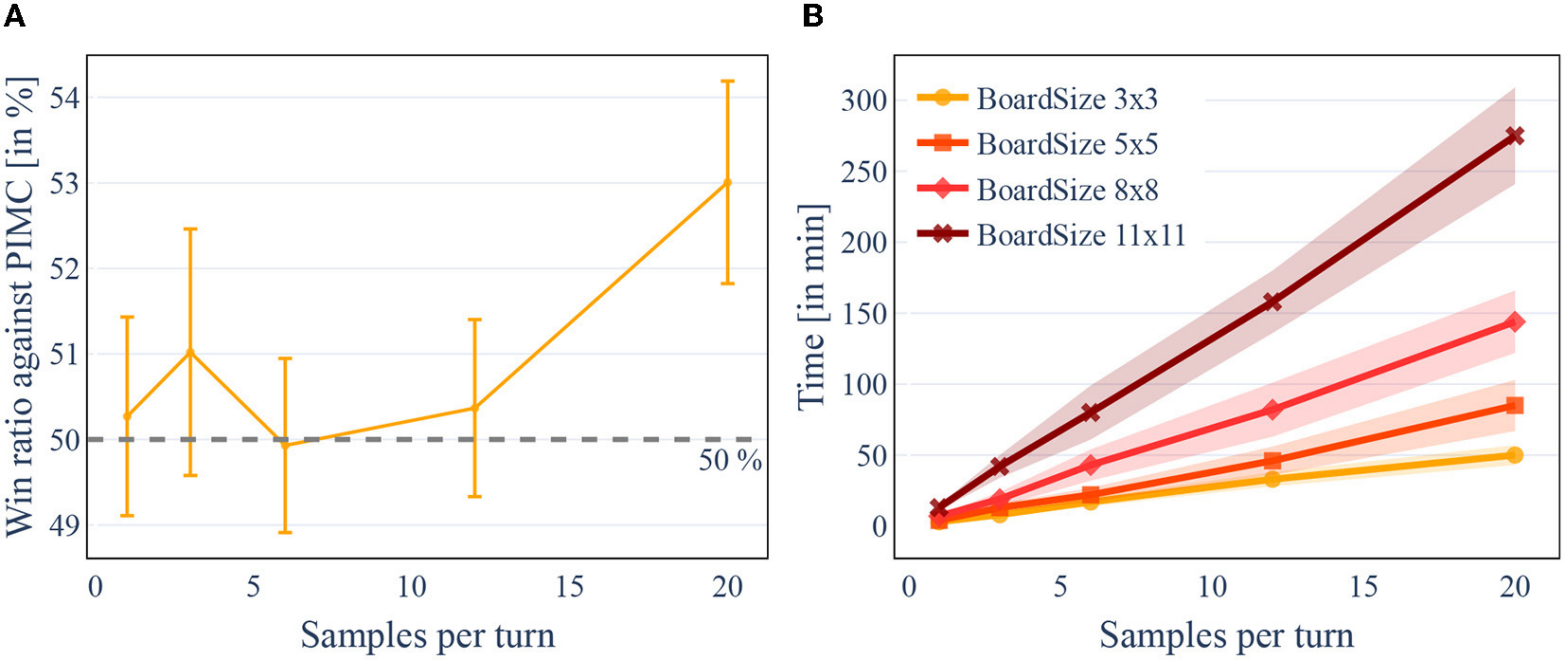
[Best viewed in color] The number of samples per turn influences the strength of the agent, but also linearly increases the time required. The size of the board also has an exponential effect on the time required. Win ratio of PC-PIMC vs. PIMC with different amounts of trees evaluated for 8 × 8 DarkHex. Elapsed time for 100 games in DarkHex, measured for different amounts of samples per turn. **(A)** Win ratio of PC-PIMC vs. PIMC with different amounts of trees evaluated for 8×8 DarkHex. **(B)** Elapsed time for 100 games in DarkHex, measured for different amounts of samples per turn.

Next, we evaluated the difference in playing strength between PIMC and PC-PIMC. For this, we played 100 games of DarkHex on an 8x8 board in a direct comparison and repeated this with different amounts of sampled states within PIMC or PC-PIMC, called “samples per turn.” In this experiment, we used 3 models, each trained over 25 epochs (around 10.2 M sample). With one MCTS tree per turn, both approaches are as expected about equally strong, since here no combination of policies takes place and the difference between policy and value does not matter. The evaluation over an increasing number of MCTS trees per turn can be seen in Figure 6. Figure 6A shows us that for small sample sizes it makes little difference whether we use PIMC or PC-PIMC. Only at 20 samples per turn we can identify a clear difference between the two.

When we increase the sample size, two things can be noticed. First, the training time and the evaluation time increases, as can be seen in Figure 6B. This is primarily due to the evaluation within the search increasing linearly with the number of samples. On an 8 × 8 board the evaluation of a game takes 3.6 s per sample, i.e., about 10.8 s for a sample size of 3. Increasing the sample size therefore increases the time needed per turn. Second, the number of samples affects the playing strength of our agents.

However, the difference in playing strength with increasing number of samplings also has a strong effect on the time the agent needs per turn. In reality there are situations where the time an agent has per decision is limited. This tends to be the practice especially in competitive situations and unlimited time tends to be the exception. We have investigated how a time limit per turn affects the agents' strength, relative to the number of samples per turn. The results are shown in Table 6. Here we choose DarkHex models with 6, 12, and 20 samples per turn, due to the time needed for them to decide on a move. Our current implementation needs around 800 ms per move when calculating six samples, 1,700 ms for 12 samples per turn and about 3,200 ms for 20 samples, if we select a fixed depth of 800 nodes per tree/sample. Using a time limit we did not reduce the amounts of samples; instead we reduced the amount of nodes per sample, i.e., while playing with a time limit of 800 ms, the agents with 20 samples have 20 relatively shallow trees.

**Table 6:** The number of samples per turn plays an important role in situations where time is limited.

**Samples per turn**	**Sampling Algorithm**	**Time per turn**			
		**800 ms**	**1,600 ms**	**3,200 ms**	**800 nodes per sample**
6	PIMC	32	46	51	30
	PC-PIMC	37	43	46	29
	TSL+PIMC	42	46	52	43
	TSL+PC-PIMC	41	51	54	44
12	PIMC	36	45	67	43
	PC-PIMC	35	42	71	41
	TSL+PIMC	41	49	71	51
	TSL+PC-PIMC	39	–	74	49
20	PIMC	11	33	60	67
	PC-PIMC	18	36	62	65
	TSL+PIMC	12	41	75	72
	TSL+PC-PIMC	18	38	81	78

If there is enough time, a higher number of samples will lead to better results in DarkHex. The table shows the win probabilities of agents with different time per move and samples per turn against our TSL+PC-PIMC agent with 12 samples per turn and a time limit of 1,600 ms per move. Each agent played 100 games against the benchmark agent in DarkHex with board size 5 × 5. The column “800 nodes” describes an agent that has unlimited time to reach 800 nodes per tree for each sample.

If we look at the results which are achieved without a time limit, we can see a clear increase in the playing strength with an increasing number of samples. Also, even if it should be clear, increasing the time per move, i.e., increasing the depth of the samples, means improving the playing strength. Also we see that many samples at a low time limit is not useful, so the agent with 20 samples loses at 1,600 ms against our agent with 12 samples at 1,600 ms. This suggests that the depth of the trees is as relevant as the number of trees.

In Stratego the results were slightly different: Here we saw clearly that agents with smaller number of samples, like 3, perform better than agents with 6 or 12 samples. Only with larger sample sizes (20 samples) there is a turnaround in performance. This problem can be explained by strategy fusion. The result of a combined policy of three samples deviates less strongly from the three samples, than a combined policy with six samples. If the sampled states are too different, AlphaZe∗∗ tries to find a policy that works in all these situations, which is not always possible. Only with a large number of samples can the combined policy better represent the information set and the errors that occur are less pronounced. However, we do not solve the problem of strategy fusion by using more samples even with infinite samples. The problem is that we try to find a policy which works in every state of our information set (see Section 4.3).

We also test our algorithm on chess and compared it with CrazyAra's (Czech et al., 2020) performance when trained under the same conditions. No difference could be found here. This is not surprising, since AlphaZe∗∗ behaves identically to AlphaZero/CrazyAra on perfect information games. We also were able to train AlphaZe∗∗ on the regular perfect information version of DarkHex, i.e., Hex, effectively.

For DarkHex, we further tested to what extent we can take an already-trained Hex model as a starting point, similar to TSL, and if this cannot even harm our training. Here it has been shown that the behavior is identical to that of TSL. Both TSL and the use of a Hex model do not perform well in DarkHex directly. Only by training further and adapting to DarkHex, these models make the transfer and are competitive. This is consistent with our findings in Figure 5A, where TSL agents also perform well only after switching to another strategy.

## 10. Discussion

### 10.1. Using TSL and PC-PIMC

The results in Figures 4, 5 show that TSL-supported methods are able to learn the game, but fail in the beginning of training when compared with methods that rely directly on sampling. Only after switching from TSL to pure sampling, the method that relies on TSL achieves comparable results and manages to produce slightly better results in our tests on DarkHex 5 × 5 and 11 × 11 as well as Barrage Stratego. This suggests that the agents are able to transfer from the TSL-supported training to the game situation. However, the difference between TSL methods and pure sampling methods decreases with training, suggesting that this transfer is lost during training. Furthermore, our experiments on the smaller 3 × 3 board seem to show that TSL does not directly improve game performance when we train both agents to convergence. Degradation from the use of TSL can occur when agents are not given time without TSL to adapt, i.e., a period in which TSL-supported agents can train without TSL. In summary, the use of TSL can only lead to improvement in cases where games are so complex that we do not train to the optimum. However, there is also a risk of achieving the same or worse results. If we already have a model for perfect games with the same or similar mechanics (in our case Hex for DarkHex), using this as a starting point can lead to agents being able to learn a little faster.

The results regarding PC-PIMC and PIMC in Figure 6A suggest preferring PC-PIMC over PIMC due to its better performance at higher amount of samples. However, since the difference in playing strength between PIMC and PC-PIMC is rather small like in Figure 5B, we see PIMC as a good alternative to PC-PIMC as a useful sampling method within AlphaZe∗∗. Fundamental problems of PIMC, as mentioned in Section 4.3, still remain in PC-PIMC, resulting in AlphaZe∗∗ occasionally playing unreliably and changing strategies frequently between moves. These problems increase with the set of possible information states within a game, i.e., the set from which we draw samples, but can be reduced by smarter sampling techniques.

By considering multiple samples to obtain a common policy, in addition to the problems already mentioned, the trade-off between diversity and accuracy arises, in our case the trade-off between the number of samples and the depth of the trees computed in the samples. This problem is not new, as described by Liu et al. (2019) and is determined by various parameters. A possible improvement that we have not considered in this work would be the parallel calculation of the samples per turn.

### 10.2. Robustness to rule changes

Our experiments regarding robustness to changes in the environment, Table 5, indicate that AlphaZe∗∗ is able to handle rule changes in the form of the subvariants quite well. It increases its win ratio in 9 out of 12 cases. Exceptions are Asmodeus and Vixen: these two agents using a version of the shortest path algorithm can improve with the additional information too, in some subvariants even more successfully than our approach.

### 10.3. AlphaZe∗∗ compared to P2SRO and DeepNash

When matched against a P2SRO^8^ agent that was trained for 820,000 episodes by McAleer et al. (2020), AlphaZe∗∗ fares worse. While P2SRO plays reasonable strategies, AlphaZe∗∗ does not necessarily, as mentioned above. This results in a win rate of 4–16% against P2SRO, depending on the amount of nodes used in the planning. Interestingly, the same problem also appears against the other bots but less severe since the amount of possible information states is smaller and we play similar or identical strategies more often.

As discussed, Table 5 examines the strength of play when both agents start with more information. We have integrated our new “Blocked Fields” variant for P2SRO, but the other custom variants appear to be difficult to implement. Here we notice a great improvement of AlphaZe∗∗ against P2SRO compared to regular Stratego, with AlphaZe∗∗ winning ~40% of the games. This strengthens our claim that AlphaZe∗∗ appears to be more robust to rule changes than other AI-approaches.

In summary, P2SRO performs better in Barrage Stratego, but AlphaZe∗∗ is easier to modify while retaining the benefits of an explicit search algorithm and is more robust to rule changes or adaptations to the environment.

In 2022, Deepmind introduced DeepNash (Perolat et al., 2022), a new approach that can play Stratego at a previously unknown level. The approach is also capable of learning the game from scratch by playing itself, without using any human knowledge, to a level where a Nash equilibrium is achieved for the game. While the training is similar to our idea, the big difference between the approaches are the algorithms used. Our approach is based on a model-based approach with a dedicated search algorithm, while DeepNash is based on a game-theoretic, model-free, search-free algorithm called Regularized Nash Dynamics, which they introduced in their work.

A direct comparison with DeepNash was not feasible thus far, partly due to the fact that DeepNash is, at the time of this work, not open source and a reimplementation seemed very time-consuming to us. However, it can be assumed that DeepNash will also be superior to our agent. This can also be assumed looking at their results against existing Stratego bots, like Asmodeus or Celsius in Stratego, where they win over 98% of their games. AlphaZe∗∗ is not as strong in a direct comparison with P2SRO, losing over 80% of its games against it, or presumably DeepNash, which seems to be much stronger. We show that our adaption of AlphaZero, i.e., a model-based RL approach, can be robust and score similar win rates as P2SRO against our baselines Celsius, Asmodeus and Vixen, but cannot reach the current state-of-the-art.

## 11. Conclusion

In this work, we presented a framework to successfully employ methods initially developed for perfect information games on imperfect ones. AlphaZe∗∗ is based on the combination of deep neural networks with tree search. We replaced the normally used MCTS with our own Policy Combining Perfect Information Monte-Carlo as well as our extension called TrueSight Learning. We demonstrated that our framework is able to learn the game of Barrage Stratego as well as DarkHex and improve the quality of moves over the learning process. Most importantly, we demonstrated that one can use known reinforcement learning frameworks, like Silver (2017) and Czech et al. (2020), on imperfect information games while only replacing the tree search. We show that despite the general concern that techniques originally developed for perfect information environments are doomed to failure on imperfect games, they can represent a serious alternative.

AlphaZe∗∗ is a surprisingly strong baseline in stark contrast to common beliefs. In its core, AlphaZe∗∗ is the combination of the two known methods, AlphaZero and (PC-)PIMC, staying as close as possible to AlphaZero. In doing so, we introduce as few new parameters into the algorithm as possible in order to maintain AlphaZero's simplicity and improve its generalizability. That is, it is the simplest extension of AlphaZero we can imagine. Compared to heuristics and oracle-based approaches, AlphaZe∗∗ can easily deal with rule changes, e.g., when more information than usual is given, and drastically outperforms other approaches in this respect. This flexibility provides several interesting avenues for the future.

One possible improvement is a more intelligent sampling method, e.g., using models to predict probabilities based on beliefs and modeling the opponent, rather than using a uniform distribution or ignoring the opponent. Another idea is to integrate the setup phase into the Stratego environment to allow a neural network to learn the placement of pieces. Furthermore, other alternatives to PC-PIMC such as Information Set Monte-Carlo (Cowling et al., 2012, 2015) or combining our PC-PIMC idea with other adaptations of MCTS should be explored. Further, there are other games worth investigating. Examples could be Poker or 2048, which are both imperfect information games with non-deterministic elements. Of course, these elements would also have to be sampled, which would pose completely new challenges.

## Data availability statement

The dataset is generated via self play. All of our repositories are publicly available on GitHub at: AlphaZe∗∗ is part of the CrazyAra repository (https://github.com/QueensGambit/CrazyAra). The original fork can be found at https://github.com/BluemlJ/AlphaZe. The code for Stratego and DarkHex are adaptations of the Open_Spiel repository (https://github.com/deepmind/open_spiel). Our adaptations are available in their own repository (https://github.com/BluemlJ/open_spiel_stratego_hex). The data used to generate starting positions for Stratego and Barrage Stratego can be found at: https://www.gravon.de/strados2/files/.

## Author contributions

This work was initially started by JB in form of a Master's thesis, supervised by JC and KK. The thesis was not published in any form, and its main goal was the development of PC-PIMC and its evaluation for the board game of Barrage Stratego. After the thesis, further experiments, like the subvariants as well as the evaluation on DarkHex were added. The paper was mainly written by JB with support and revision of JC and KK. All implementations were based or use the CrazyAra framework by JC and written by JB with continuous support and help of JC. All authors contributed to the article and approved the submitted version.

## Footnotes

^1^The ** indicate that AlphaZe∗∗ is a version of AlphaZero for imperfect information games.

^2^Stratego is a trademark of Jumbo Games, and is used in this publication for informational purposes only.

^3^The code of AlphaZe∗∗ is part of the CrazyAra framework (https://github.com/QueensGambit/CrazyAra).

^4^ https://www.gravon.de/strados2/files/

^5^More information on the FEN notation can, e.g., be found at: https://en.wikipedia.org/wiki/Forsyth-Edwards_Notation.

^6^Some of the resulting models, used for our evaluation, can be found at: https://github.com/QueensGambit/CrazyAra/releases/tag/1.0.2.

^7^ https://github.com/braathwaate/strategoevaluator

^8^ https://github.com/JBLanier/pipeline-psro
